# Perceived oral health and disease experience among adults in Sierra Leone: An exploratory study

**DOI:** 10.4102/jphia.v16i1.1385

**Published:** 2025-08-28

**Authors:** Swapnil G. Ghotane, Ahmed Al-Baiyaa, Stephen J. Challacombe, Patric Don-Davis, David Kamara, Jennifer E. Gallagher

**Affiliations:** 1 Department of Women & Children’s Health, Faculty of Life Sciences and Medicine, King’s College London, London, United Kingdom; 2 Centre for Host Microbiome Interactions, Faculty of Dentistry, Oral & Craniofacial Sciences, King’s College London, London, United Kingdom; 3 College of Medicine and Allied Health Sciences, Freetown, Sierra Leone; 4 Department of Oral Health, Connaught Hospital, Freetown, Sierra Leone

**Keywords:** Sierra Leone, dentistry, adults, perceived oral health, oral disease, ICDAS, PUFA, oral health survey, adult dental health survey

## Abstract

**Background:**

Oral health often receives low priority in fragile countries like Sierra Leone (SL), which have constrained health systems and resources.

**Aim:**

To explore both normative and perceived oral health needs of adults in SL to guide strategies for the development of future oral health programmes and services.

**Setting:**

This study was conducted across all four regions of SL.

**Methods:**

This study utilised a self-completion questionnaire exploring access to dental care, oral hygiene practices, diet, risk behaviours, general and dental health and oral health-related quality of life. Clinical examinations used the International Caries Detection and Assessment System (ICDAS) and the PUFA (pulp, ulcer, fistula, abscess) Index, among other tools. Descriptive statistics summarised key variables, while bivariate analyses explored associations using STATA and Statistical Package for Social Sciences (SPSS).

**Results:**

One hundred and sixty one participants aged between 19 years and 70 years (mean = 35.6 years) completed the questionnaire survey. Two-thirds (75%, *n* = 121) reported having ‘never been to a dentist’, 40% (*n* = 62) experiencing toothache and 68% (*n* = 82) consuming one or more sugary items at least once a day, particularly males. Urban participants reported good dental health (63%), while 17% – 29% of rural participants reported significant impacts on daily life due to oral health issues. Of the 45 participants who underwent a clinical examination, 84% (*n* = 38) had cavitated dental caries into dentine with over five heavily diseased teeth on average (D_5–6_MFT = 5.2).

**Conclusion:**

This survey suggests a high level of perceived need and untreated dental disease among participating adults, limited dental care access, notable sugar consumption and significant impacts on quality of life.

**Contribution:**

This study highlights the necessity for a national-level adult dental health survey to better inform the planning of oral health services in support of adults in SL.

## Introduction

Symptoms of oral disease, including pain, difficulty in swallowing, speaking and appearance, have been reported to severely impact the living experience of individuals, resulting in loss of social and work productivity.^1^ However, a considerable disparity has been shown between low-, middle- and high-income countries with regard to the perception of such problems and the way that their populations cope with their effects.^2^ Low-income countries, such as Sierra Leone (SL) in West Africa, may regard dental caries and periodontal disease, which are one of the key contributors to the global burden of chronic diseases,^3^ as of less worth when compared with major infectious diseases carrying greater mortality risks such as Ebola and more recently the coronavirus disease 2019 (COVID-19) pandemic.^4^ High-income countries are increasingly challenged by the rising cost of dental services, the growing complexity of oral health needs in ageing populations who retain more natural teeth and a significant burden of unmet dental care, particularly among marginalised communities.^5^ These disparities in the perception and prioritisation of oral health problems across income settings are well documented, with evidence indicating that low-income countries such as SL often underprioritise oral diseases due to constrained resources, competing health burdens and limited service infrastructure.^6,7^

Sierra Leone is a fragile state^8^ with a weak health system.^9^ This has accentuated the limited human resources for oral health with approximately only 10 whole-time equivalent dentists and a similar number of dental care professionals (including dental therapists, dental assistants and oral health promoters) available for a population in excess of seven million.^10,11^ There is a marked dearth of evidence on oral health of adults in SL. A descriptive study in 1975 highlighted poor public awareness of oral hygiene alongside systemic deficiencies, including a shortage of dental personnel and limited government support.^12^ Subsequently, a clinical study conducted in the 1990s reported 85% of 35–44 year olds having either decayed (D), missing (M) or filled (F) teeth (T) with an average DMFT value of 5.3.^13^ It further reported that 53% of adults in SL had deep periodontal pockets,^13^ rates higher than those observed in many other African countries at the time.^14^ Similarly, a 1995 study in a remote village of SL-documented moderate levels of dental disease among adults, exacerbated by the absence of dental services and restricted access to care.^15^ Collectively, these findings underscored the need for current data on the oral health of Sierra Leonan adults.

Current global health policy^16^ advocates for Universal Health Coverage (UHC)^17^ and integrating oral health into UHC presents a unique opportunity to improve oral health outcomes and reduce inequalities.^18^ This aligns with the Regional Oral Health Strategy 2016–2025 for Africa, which proposes a multi-faceted approach of oral health integration into national noncommunicable diseases (NCD) programmes, strengthening oral health surveillance and research and enhancing oral health workforce capacity.^19^ However, evidence suggests that merely expanding traditional health services is insufficient; rather, a revolution in health systems is needed, including greater investment in research and data collection to inform evidence-based decision-making.^16,20^ Sierra Leone has therefore developed and approved a new oral health strategy,^21^ informed by collaborative research.^4,11,22^

The King’s Sierra Leone Partnership (KSLP) is a collaborative initiative between King’s Health Partners in the United Kingdom (UK) and partners in SL, aimed at strengthening the healthcare system in SL with focus on improving healthcare delivery, training healthcare workers and building local capacity.^23^ King’s Sierra Leone Partnership supported researchers at King’s College London (KCL) to undertake the first national child dental health survey of schoolchildren in SL (CDHS-SL) to explore normative and perceived oral health needs and inform the vision of improving oral healthcare delivery and capacity strengthening.^22^ Given the young SL population (almost 41% under the age of 15 years in 2017)^24^ and the feasibility of conducting a research on a national scale, the focus of the epidemiological survey was on child oral health.^22^ The opportunity, however, was taken to examine a convenience sample of adults considering the challenge of seeking a representative sample of adults, to provide timely insights into SL’s adult oral health status. In addition, addressing this evidence gap was essential, as no current data exist and the few available studies were outdated, small in scale and consistently point to a substantial documented burden of dental and periodontal disease among adults of SL.^13,15^ Hence, the objective of this study was to report the normative and perceived oral health needs of adults in SL in parallel with the national survey of schoolchildren.

## Research methods and design

### Study design

This study was conducted in 2017 alongside the fieldwork for the national CDHS.^22^ The research drew on two internationally accepted epidemiological methodologies including the World Health Organization (WHO) pathfinder survey method,^25^ and the UK Adult Dental Health Survey (ADHS).^26^

### Setting

This study was conducted across all four regions (areas) of SL as follows: Northern (Makeni City and Libeisaygahun), Southern (Selenga, Kori and Bo Town), Eastern (Kenema City and Hangha village) and Western (Waterloo Lumpa and Regent).

### Study population and sampling strategy

The aim was to have at least 50 adults from each of the four regions of SL in line with the WHO sampling method,^25^ described in an earlier paper.^22^ In summary, adults who were present at the school site on the day of the CDHS-SL were provided with a ‘pictorial information and consent’ (PIC) sheet regarding the study.^27^ The PIC sheet informed participants about a basic oral examination with a trained dental professional, following a short self-completion questionnaire. Where necessary, this information was reiterated by an SL team member in their preferred local language (mainly in Krio, Mende or Temne).

### Parameters and their measurements

#### Clinical data

In relation to the clinical epidemiological examination, the ‘International Caries Detection and Assessment System (ICDAS)’ tool was used to detect untreated decay (D), filled (F) and missing (M) teeth^28^; PUFA (pulp, ulcer, fistula, abscess) index to detect conditions arising due to untreated dental caries and periodontal health assessment (calculus) from the UK ADHS^26^, and finally, oral mucosal lesions, tooth wear, fluorosis and denture status were assessed using the WHO criteria.^25^

#### Socio-demographic and behavioural data

The self-completion questionnaire was adapted locally for SL, from the WHO^25^ and the UK ADHS^26^ and included items across six themes as follows: (1) access and barriers to receive dental care; (2) oral hygiene practices including tooth brushing tools and frequency; (3) diet including frequency of consumption of certain food and drinks; (4) risky health behaviours, such as smoking and drinking alcohol and their frequency; (5) history of perceived (self-rated) dental and general health and (6) oral health-related quality of life (OHrQoL) including types of dental problems, difficulties experienced with aesthetics, functioning, work and everyday life.

### Data collection

Participants were provided with the self-completion questionnaire and assisted by a local SL team member, if required. The internal consistency of the self-completion questionnaire was evaluated using Cronbach’s alpha, yielding a coefficient of 0.827, indicating good reliability.

The oral examination was carried out by a trained and calibrated dentist supported by the local SL team members who acted as a recorder to capture data in a paper format.^22^ All examinations were conducted under natural daylight using a standardised portable chair, appropriate personal protection equipment (PPE) and disposable clinical instruments. Examinations were performed by a single calibrated examiner, with intra-rater reliability (kappa = 0.61) confirming substantial agreement. As a token of appreciation, each adult was provided with toothpaste following their participation in the study.

### Data analysis

Data were analysed using STATA and Statistical Package for Social Sciences (SPSS).^29,30^ Descriptive statistics were carried out to report the demographic characteristics of the sample. Frequencies and proportions were calculated for all questionnaire items. Bivariate analyses using chi-square tests were performed to explore associations between questionnaire items and demographic variables including sex, age groups (18–24 years, 25–34 years, 35–44 years and 45+ years), area (urban; rural) and region (east, north, south and west). For participants who underwent clinical examination, univariate and bivariate analyses were conducted using parametric tests (*t*-test, analysis of variance [ANOVA]) and non-parametric tests (Mann–Whitney U, Kruskal–Wallis) to assess associations between demographic factors and clinical indicators, including dental caries experience (DMFT), PUFA, oral mucosal lesions, tooth wear, dental fluorosis and denture status.

### Ethical considerations

Ethical approval for the study was received from King’s Research Ethics Committee (HR-15/16-1951) and the Sierra Leone Ethics and Scientific Review Committee (RESCMR-16/17-1951). All procedures performed involving human participants were in accordance with the ethical standards of the institutional research committee and with the 1964 Helsinki Declaration and its later amendments or comparable ethical standards. Participants were informed that taking part in the study was entirely voluntary, and there was no incentive on offer. If they agreed to participate, a signed consent (through ‘pictorial information and consent’ [PIC] sheet) 27 was obtained. Participants were informed that clinical examination would only be conducted at that site after the examination of school children had been completed. All data were anonymised using unique codes that ensured privacy and confidentiality of all participants. Data were securely stored, and access was restricted to the research team.

## Results

### Demographic characteristics of participants

One hundred and sixty-one participants aged 19 years to 70 years (mean age = 35.6 years), predominately female (*n* = 95; 60%), completed the questionnaire survey, and 45 underwent a clinical epidemiological examination. Among participants who had a clinical examination, 64% (*n* = 29) were female. The proportion of participants from rural areas was 44% (*n* = 71) and most participants who provided information on ethnicity were from the ‘Mende’ tribe (26%; *n* = 41) (Table 1).

**TABLE 1:** Demographic characteristics of participants: questionnaire survey (*n* = 161) and clinical examination (*n* = 45).

Demographic items	Total participants for the questionnaire		Clinical examination conducted	
	%	*n*	%	*n*
**Region**				
East	31	50	12	19
North	16	26	0	-
South	42	67	12	18
West	11	18	4	8
Missing	0	0	0	0
**Sex**				
Female	60	95	18	29
Male	40	64	10	16
Missing	1	2	0	-
**Age groups (years)**				
18–24	12	19	9	4
25–34	33	54	36	16
35–44	36	58	44	20
45+	16	25	11	5
Missing	3	5	0	0
**Area**				
Urban	56	90	10	16
Rural	44	71	18	29
Missing	0	0	0	0
**Disability**				
No	89	143	26	42
Yes	7	12	1	2
Missing	4	6	1	1
**Ethnicity**				
Mende	26	41	18	28
Temne	8	13	5	8
Fullah	4	6	3	5
Others	7	11	2	4
Missing	56	90	0	-

**Total**	**100**	**161**	**28**	**45**

### Access to dental care

Three-quarters of participants had ‘never been to a dentist’ (75%; *n* = 121), and one in five (21%; *n* = 33) only had visited a dentist because of ‘problems with their teeth’. Routine check-up attendance was less commonly practised in this group and reported by only 1% (*n* = 2) of participants (Table 2). Access to dental care varied by age, with a borderline association (likelihood ratio [LR] = 17.70, *p* = 0.04); participants aged 35–44 years were least likely to report ‘never been to a dentist’ (Table 3).

**TABLE 2:** Oral health behaviours, self-reported health and quality of life by demographic characteristics of all survey participants (*n* = 161) and clinical examination subgroup (*n* = 45).

Questionnaire themes	Items	Item categories	Sex				Age groups (years)								Area				Region								Clinical examination subgroup (*n* = 45)	
			Female		Male		18–24		25–34		35–44		45+		Urban		Rural		East		North		South		West			
			%	*n*	%	*n*	%	*n*	%	*n*	%	*n*	%	*n*	%	*n*	%	*n*	%	*n*	%	*n*	%	*n*	%	*n*	%	*n*
**Access**	Visit to a dentist	Never been to a dentist	80	76	69	44	89	17	83	45	69	40	56	14	74	67	76	54	60	30	85	22	82	55	78	14	71	32
		Go only when I have trouble with teeth	17	16	25	16	11	2	9	5	29	17	36	9	19	17	23	16	32	16	15	4	13	9	22	4	24	11
		Went for check up	0	-	3	2	0	-	2	1	0	-	4	1	2	2	0	-	4	2	0	-	0	-	0	-	0	-
**Oral hygiene**	Brushing frequency	Twice a day	68	65	70	45	79	15	67	36	62	36	84	21	70	63	68	48	60	30	73	19	72	48	78	14	73	33
		Once a day	14	13	19	12	5	1	15	8	10	6	8	2	12	11	10	7	16	8	12	3	15	10	22	4	13	6
		Thrice a day or more	16	15	11	7	11	2	13	7	26	15	8	2	13	12	21	15	24	12	12	3	12	8	0	-	11	5
		Never	1	1	0	-	5	1	6	3	2	1	0	-	4	4	1	1	0	-	4	1	0	-	0	-	0	-
	Brushing tool	Toothbrush	98	93	95	61	100	19	96	52	95	55	100	25	98	88	96	68	94	47	100	26	97	65	100	18	89	40
		Toothpick	1	1	0	-	0	-	0	-	2	1	0	-	0	-	1	1	0	-	0	-	2	1	0	-	2	1
		Other	0	-	3	2	0	-	4	2	3	2	0	-	2	2	3	2	4	2	0	-	0	-	0	-	4	2
	Use of toothpaste	Yes	95	90	91	58	95	18	94	51	90	52	96	24	92	83	94	67	94	47	92	24	91	61	100	18	89	40
		No	3	3	6	4	0	-	4	2	7	4	4	1	7	6	1	1	4	2	8	2	5	3	0	-	4	2
**Diet**	Sweets (e.g. chocolates)	Once a day or less	91	85	82	50	95	18	83	44	93	52	79	19	86	77	90	60	83	38	85	22	90	60	94	17	89	40
		Twice or three times a day	0	-	5	3	0	-	2	1	2	1	4	1	2	2	2	1	4	2	0	-	2	1	0	-	2	1
		Four times or more	1	1	0	-	5	1	15	8	5	3	17	4	12	11	9	6	0	-	4	1	0	-	0	-	0	-
	Cakes and/or biscuits	Once a day or less	87	82	83	52	84	16	83	45	88	50	83	20	86	77	86	59	85	41	85	21	91	61	67	12	85	38
		Twice or three times a day	4	4	4	4	5	1	7	4	5	3	0	-	4	4	6	4	4	2	12	3	0	-	17	3	4	2
		Four times or more	1	1	2	1	11	2	9	5	7	4	17	4	10	9	9	6	0	-	0	-	0	-	11	2	2	1
	Fizzy drinks	Once a day or less	91	86	87	54	95	18	89	47	91	53	79	19	90	81	88	61	86	41	96	25	91	61	83	15	87	39
		Twice or three times a day	1	1	3	2	0	-	0	-	2	1	8	2	1	1	3	2	4	2	0	-	2	1	0	-	2	1
		Four times or more	4	4	5	3	5	1	11	6	7	4	13	3	9	8	9	6	6	3	0	-	3	2	11	2	4	2
	Fruit juice	Once a day or less	90	85	87	54	90	17	85	45	95	54	79	19	88	79	90	61	87	41	89	23	90	60	89	16	87	39
		Twice or three times a day	2	2	2	1	0	-	4	2	2	1	4	1	1	1	4	3	2	1	4	1	0	-	11	2	7	3
		Four times or more	0	-	0	-	11	2	11	6	4	2	17	4	11	10	6	4	0	-	0	-	0	-	0	-	0	-
	Water	Once a day or less	2	2	0	-	0	-	2	1	2	1	0	-	0	-	3	2	0	-	4	1	0	-	6	1	2	1
		Twice or three times a day	4	4	3	2	0	-	4	2	7	4	0	-	4	4	3	2	6	3	4	1	3	2	0	-	0	-
		Four times or more	85	80	89	56	100	19	94	51	91	52	100	24	96	86	94	65	85	41	92	24	84	56	94	17	89	40
	Fruits	Once a day or less	10	9	24	15	26	5	13	7	17	10	8	2	14	13	16	11	20	10	23	6	5	3	28	5	25	11
		Twice or three times a day	71	67	67	40	47	9	72	39	69	40	80	20	67	60	73	52	76	38	54	14	84	56	22	4	58	26
		Four times or more	18	17	8	5	26	5	15	8	14	8	12	3	19	17	11	8	4	2	23	6	8	5	50	9	13	6
**Risk behaviours**	Smoking	Never	98	93	67	43	94	17	94	48	88	50	72	18	89	76	87	62	78	39	92	24	90	60	83	15	87	39
		Tried sometimes or occasionally	0	-	6	4	6	1	0	-	2	1	8	2	2	2	3	2	4	2	0	-	2	1	6	1	0	-
		Regularly	1	1	20	13	0	-	6	3	11	6	20	5	8	7	10	7	12	6	8	2	6	4	11	2	9	4
	Alcohol	Never	95	90	64	41	100	18	88	45	83	48	68	17	85	73	85	60	74	37	92	24	81	54	100	18	80	36
		Tried sometimes or occasionally	2	2	16	10	0	-	6	3	7	4	20	5	9	8	6	4	18	9	0	-	5	2	0	-	4	2
		Regularly	2	2	16	10	0	-	6	3	10	6	12	3	6	5	10	7	4	2	8	2	12	8	0	-	11	5
**History of general and dental health**	Overall general health	Good or very good	85	81	74	47	79	15	85	46	77	44	80	20	82	73	80	57	72	36	77	20	96	64	56	10	80	36
		Fair	13	12	23	15	21	4	15	8	19	11	16	4	16	14	18	13	24	12	23	6	3	2	39	7	16	7
		Bad or very bad	1	1	3	2	0	-	0		4	2	4	1	2	2	1	1	4	2	0	-	0	-	6	1	4	2
	Overall dental health	Good or very good	57	54	47	30	74	14	52	28	49	28	60	15	63	57	41	29	42	21	54	14	60	40	61	11	47	21
		Fair	35	33	45	29	26	5	41	22	40	23	32	8	31	28	49	34	50	25	35	9	34	23	28	5	40	18
		Bad or very bad	8	8	6	4	0	-	7	4	11	6	8	2	6	5	10	7	8	4	8	2	6	4	11	2	13	6
**Oral health related quality of life**	Dental problems in last 3 months	Toothache	37	35	41	26	16	3	41	22	45	26	36	9	30	27	49	35	42	21	31	8	36	24	50	9	44	20
		Broken tooth	1	1	13	8	0	-	2	1	9	5	12	3	7	6	4	3	14	7	4	1	2	1	0	-	4	2
		Sensitivity	4	4	3	2	5	1	7	4	2	1	0	-	4	4	3	2	4	2	0	-	6	4	0	-	11	5
		Bleeding/swollen gums	2	2	5	3	0	-	6	3	3	2	0	-	4	4	1	1	6	3	0	-	3	2	0	-	4	2
		Bad breath	0	-	2	1	0	-	2	1	0	-	0	-	1	1	0	-	2	1	0	-	0	-	0	-	0	-
	Teeth aesthetics and/or appearance	Satisfied or very satisfied	51	48	42	27	84	16	40	21	40	23	64	16	50	45	46	32	38	19	58	15	52	35	44	8	40	18
		Neutral	35	33	36	23	16	3	34	18	48	28	16	4	33	30	37	26	40	20	31	8	36	24	22	4	42	19
		Dissatisfied or very dissatisfied	14	13	22	14	0	-	26	14	12	7	20	5	17	15	17	12	22	11	12	3	10	7	33	6	16	7
	Difficulty in eating	Not at all	58	55	47	30	74	14	49	26	50	29	60	15	61	55	46	32	36	18	73	19	60	40	56	10	47	21
		A little bit	22	21	33	21	26	5	28	15	26	15	24	6	27	24	26	18	40	20	19	5	21	14	17	3	33	15
		A fair amount or a lot	19	18	20	13	0	-	23	12	24	14	16	4	12	11	29	20	24	12	8	2	18	12	28	5	18	8
	Difficulty in Speaking	Not at all	63	60	59	38	84	16	57	30	55	32	76	19	70	63	53	37	52	26	73	19	66	44	61	11	60	27
		A little bit	20	19	23	15	16	3	23	12	22	13	20	5	21	19	21	15	22	11	19	5	19	13	28	5	24	11
		A fair amount or a lot	16	15	17	11	0	-	21	11	22	13	4	1	9	8	26	18	26	13	8	2	14	9	11	2	13	6
	Difficulty in cleaning teeth	Not at all	61	58	52	33	79	15	55	29	52	30	64	16	67	60	47	33	44	22	73	19	63	42	56	10	51	23
		A little bit	21	20	33	21	21	4	26	14	26	15	28	7	24	22	27	19	32	16	19	5	21	14	33	6	31	14
		A fair amount or a lot	17	16	16	10	0	-	19	10	22	13	8	2	9	8	26	18	24	12	8	2	15	10	11	2	16	7
	Difficulty in relaxing and/or sleeping	Not at all	62	59	55	35	84	16	55	29	54	31	68	17	67	60	52	36	48	24	73	19	63	42	61	11	58	26
		A little bit	19	18	33	21	16	3	25	13	26	15	28	7	24	22	25	17	30	15	19	5	24	16	17	3	24	11
		A fair amount or a lot	17	16	13	8	0	-	21	11	19	11	4	1	9	8	23	16	22	11	8	2	12	8	17	3	13	6
	Felt upset and/or irritated	Not at all	61	58	56	36	84	16	55	29	55	32	64	16	67	60	51	36	48	24	69	18	64	43	61	11	58	26
		A little bit	19	18	30	19	16	3	25	13	22	13	28	7	23	21	23	16	26	13	19	5	22	15	22	4	27	12
		A fair amount or a lot	19	18	14	9	0	-	21	11	22	13	8	2	10	9	26	18	26	13	12	3	12	8	17	3	13	6
	Difficulty in smiling	Not at all	62	59	58	37	89	16	54	28	55	32	76	19	69	62	53	36	50	25	73	19	63	42	67	12	58	26
		A little bit	17	16	27	17	11	2	21	11	26	15	16	4	21	19	21	14	24	12	15	4	21	14	17	3	22	10
		A fair amount or a lot	18	17	16	10	0	-	25	13	19	11	8	2	10	9	27	18	24	12	12	3	14	9	17	3	13	6
	Difficulty in completing work	Not at all	61	58	56	36	84	16	55	29	53	31	71	17	67	60	51	36	48	24	69	18	64	43	61	11	58	26
		A little bit	23	22	30	19	16	3	28	15	28	16	25	6	24	21	29	20	28	14	23	6	22	15	33	6	29	13
		A fair amount or a lot	14	13	14	9	0	-	17	9	19	11	4	1	9	8	20	14	24	12	4	1	12	8	6	1	11	5
	Difficulty enjoying being with people	Not at all	61	58	56	36	84	16	55	29	54	31	68	17	68	61	51	35	48	24	69	18	64	43	61	11	56	25
		A little bit	20	19	30	19	16	3	23	12	28	16	24	6	22	20	26	18	28	14	19	5	22	15	22	4	29	13
		A fair amount or a lot	17	16	14	9	0	-	23	12	18	10	8	2	10	9	23	16	24	12	12	3	12	8	11	2	11	5
	Condition of teeth affected everyday life	Not at all	62	59	53	34	79	15	55	29	52	30	72	18	66	59	51	36	46	23	69	18	64	43	61	11	56	25
		A little bit or somewhat	23	22	31	20	21	4	28	15	28	16	24	6	27	24	26	18	24	12	19	5	21	14	17	3	31	14
		A fair amount or a great deal	14	13	16	10	0	-	17	9	21	12	4	1	8	7	23	16	30	15	12	3	14	9	22	4	11	5

**TABLE 3:** Associations between oral health behaviours, self-reported health, quality of life and demographic characteristics among all survey participants (*n* = 161).

Questionnaire themes	Items	Sex (male; female)						Area (urban; rural)						Region (east, north, south, west)						Age (years) (18–24; 25–34; 35–44; 45+)					
		*χ* ^2^	*df*	*p*	LR	*df*	*p*	*χ* ^2^	*df*	*p*	LR	*df*	*p*	*χ* ^2^	*df*	*p*	LR	*df*	*p*	*χ* ^2^	*df*	*p*	LR	*df*	*p*
Access	Visit to a dentist	4.88	3	0.18	5.53	3	0.14	3.03	3	0.39	3.88	3	0.27	13.84	9	0.13	15.26	9	0.08	16.13	9	0.06	17.70	9	**0.04**
Oral hygiene	Brushing frequency	3.59	3	0.31	5.37	3	0.15	2.85	3	0.42	2.94	3	0.40	17.01	9	0.05	19.26	9	**0.02**	10.37	9	0.32	11.02	9	0.27
	Brushing tool	2.71	2	0.26	3.05	2	0.22	1.34	2	0.51	1.71	2	0.42	5.34	6	0.50	6.11	6	0.41	3.34	6	0.77	4.71	6	0.58
	Use of toothpaste	1.06	2	0.59	1.03	2	0.60	4.09	2	0.13	4.47	2	0.11	3.81	6	0.70	5.35	6	0.50	3.27	6	0.78	4.53	6	0.61
Diet	Sweets (e.g. chocolates)	5.35	2	0.07	6.32	2	**0.04**	0.56	2	0.76	0.57	2	0.75	4.00	6	0.68	4.51	6	0.61	5.41	6	0.49	5.82	6	0.44
	Cakes and/or biscuits	0.69	2	0.71	0.68	2	0.71	0.21	2	0.89	0.21	2	0.90	13.39	6	**0.04**	14.45	6	**0.03**	3.47	6	0.75	4.50	6	0.61
	Fizzy drinks	1.04	2	0.59	1.02	2	0.60	0.67	2	0.71	0.67	2	0.71	4.79	6	0.57	5.21	6	0.52	8.02	6	0.24	7.19	6	0.30
	Fruit juice	0.71	2	0.70	0.70	2	0.70	2.88	2	0.24	2.95	2	0.23	9.15	6	0.17	9.99	6	0.12	5.50	6	0.48	9.99	6	0.12
	Water	1.50	2	0.47	2.21	2	0.33	2.86	2	0.24	3.60	2	0.17	7.10	6	0.31	7.39	6	0.29	4.08	6	0.67	6.02	6	0.42
	Fruits	7.75	2	**0.02**	7.82	2	**0.02**	1.76	2	0.41	1.81	2	0.41	37.40	6	**0.01**	37.29	6	**0.01**	6.27	6	0.39	6.11	6	0.41
Risk behaviours	Smoking	26.45	2	**0.01**	29.00	2	**0.01**	0.17	2	0.92	0.17	2	0.92	3.92	6	0.69	4.37	6	0.63	11.33	6	0.08	12.63	6	0.05
	Alcohol	23.01	2	**0.01**	23.35	2	**0.01**	1.52	2	0.47	1.53	2	0.47	16.90	6	**0.01**	19.35	6	**0.01**	10.48	6	0.11	11.89	6	0.07
History of general and dental health	Overall general health	4.15	2	0.12	4.08	2	0.13	0.32	2	0.85	0.32	2	0.85	23.05	6	**0.01**	26.38	6	**0.01**	3.42	6	0.75	4.56	6	0.60
	Overall dental health	2.05	2	0.36	2.05	2	0.36	7.65	2	**0.02**	7.69	2	**0.02**	5.06	6	0.54	5.05	6	0.54	4.94	6	0.55	6.32	6	0.39
Oral health-related quality of life	Dental problems in last 3 months	4.15	2	0.12	4.08	2	0.13	0.32	2	0.85	0.32	2	0.85	23.05	6	**0.01**	26.38	6	**0.01**	3.42	6	0.75	4.56	6	0.60
	Teeth aesthetics and/or appearance	0.99	2	0.61	0.98	2	0.61	3.42	2	0.18	3.41	2	0.18	5.02	6	0.54	4.89	6	0.56	3.29	6	0.77	4.78	6	0.57
	Difficulty in eating	1.90	2	0.39	1.86	2	0.39	6.97	2	**0.03**	7.05	2	**0.03**	8.42	6	0.21	8.60	6	0.20	7.13	6	0.31	9.40	6	0.15
	Difficulty in speaking	0.34	2	0.84	0.34	2	0.84	8.71	2	**0.01**	8.75	2	**0.01**	6.70	6	0.35	6.58	6	0.36	10.79	6	0.10	14.51	6	**0.02**
	Difficulty in cleaning teeth	2.68	2	0.26	2.65	2	0.27	9.55	2	**0.01**	9.61	2	**0.01**	8.57	6	0.20	8.70	6	0.19	8.06	6	0.23	11.07	6	0.09
	Difficulty in relaxing and/or sleeping	3.80	2	0.15	3.76	2	0.15	6.65	2	**0.04**	6.64	2	**0.04**	6.35	6	0.39	6.45	6	0.37	10.20	6	0.12	13.57	6	**0.04**
	Felt upset and/or irritated	2.57	2	0.28	2.55	2	0.28	7.29	2	**0.03**	7.29	2	**0.03**	6.01	6	0.42	5.88	6	0.44	9.08	6	0.17	12.25	6	0.06
	Difficulty in smiling	1.92	2	0.38	1.90	2	0.39	7.74	2	**0.02**	7.73	2	**0.02**	4.89	6	0.56	4.85	6	0.56	12.02	6	0.06	15.01	6	**0.02**
	Difficulty in completing work	0.77	2	0.68	0.78	2	0.68	5.47	2	0.07	5.47	2	0.07	9.35	6	0.15	9.67	6	0.14	9.71	6	0.14	12.66	6	0.05
	Difficulty enjoying being with people	1.81	2	0.40	1.80	2	0.41	6.45	2	**0.04**	6.44	2	**0.04**	5.72	6	0.46	5.58	6	0.47	9.33	6	0.16	12.15	6	0.06
	Condition of teeth affected everyday life	1.57	2	0.46	1.56	2	0.46	7.57	2	**0.02**	7.59	2	**0.02**	10.54	6	0.10	10.75	6	0.10	9.57	6	0.14	12.65	6	0.05

χ^2^, Pearson Chi-square; LR, likelihood ratio; *df*, degree of freedom.

Note: Bold values indicate significance at *p* < 0.05; all analyses used two-sided tests.

### Oral hygiene practices

Most participants reported using a toothbrush (97%; *n* = 156) and toothpaste (93%; *n* = 150), while a small proportion (1%; *n* = 3) used alternative tools such as toothpicks or chewing sticks (Table 2). Almost all participants (99%; *n* = 159) reported brushing their teeth daily, with over two-thirds (69%; *n* = 111) brushing at least twice a day. Brushing frequency varied significantly by region (LR = 19.26, *p* = 0.02), with participants from the Western region most likely to report brushing twice daily (Table 3).

### Diet and risk behaviours

More than nine out of 10 (92%; *n* = 145) participants reported consuming fruit at least once a day. Twenty per cent reported consuming fizzy drinks (*n* = 22) and cakes and/or biscuits (*n* = 23) along with 17% reported consuming sweets (*n* = 11) and fruits juice (*n* = 12) at least once a day, respectively (Table 2). Male participants were more likely to report frequent consumption of sweets compared to females (LR = 6.32, *p* = 0.04), whereas higher fruit intake was more commonly reported by females (LR = 7.82, *p* = 0.02). Fruit consumption also varied significantly by region (LR = 37.29, *p* = 0.01), with participants from the Western region reporting the highest frequency of intake (four or more times per day). Similarly, regional variation was observed in the consumption of cakes and biscuits (LR = 14.45, *p* = 0.03), with the highest frequency reported in the Southern region (Table 3).

Most participants reported no history of smoking (86%; *n* = 138) or alcohol consumption (83%; *n* = 133) (Table 2). However, male participants were significantly more likely than females to report both smoking (LR = 29.00, *p* = 0.01) and alcohol use (LR = 23.35, *p* = 0.01). Regional variation was evident in alcohol consumption (LR = 19.35, *p* = 0.01), with regular use of alcohol most commonly reported in the Southern region (Table 3).

### Impact on oral health-related quality of life

Significant associations were observed between demographic factors and participants’ general health, dental health and OHRQoL (Table 3). Overall general health varied significantly by region (LR = 26.38, *p* = 0.01), with participants from the Southern region most likely to report ‘good’ or ‘very good’ health. In relation to dental health, participants residing in urban areas were more likely to report favourable ratings compared to those in rural areas (LR = 7.69, *p* = 0.02). Experience of recent dental problems also differed significantly by region (LR = 26.38, *p* = 0.01), with the highest proportions of reported ‘toothache’ found among participants from the Western and Eastern regions.

Several OHRQoL indicators were significantly associated with area (urban vs. rural). Participants from rural areas were significantly more likely to report negative impacts in items such as difficulty in speaking (LR = 8.75, *p* = 0.01), eating (LR = 7.05, *p* = 0.03), cleaning teeth (LR = 9.61, *p* = 0.01), relaxing or sleeping (LR = 6.64, *p* = 0.04), feeling upset or irritated (LR = 7.29, *p* = 0.03), smiling (LR = 7.73, *p* = 0.02), enjoying being with others (LR = 6.44, *p* = 0.04) and the overall perceived impact of dental conditions on daily life (LR = 7.59, *p* = 0.02).

Age-related differences were also observed across OHRQoL items. Difficulty in speaking was most frequently reported by participants aged 35–44 years (LR = 14.51, *p* = 0.02), while those aged 25–34 years were most likely to report difficulty in relaxing or sleeping (LR = 13.57, *p* = 0.04) and smiling (LR = 15.01, *p* = 0.02).

### Dental caries experience

Just over one-quarter of the participants who completed the questionnaire (28%; *n* = 45) underwent a clinical examination with all having dental caries experience (D_2–6_MT > 0) (Table 4). Almost four out of 10 of these participants had missing teeth, with an average of four missing teeth per adult.

**TABLE 4:** Decayed (DT), missing (MT), filled (FT) teeth and dental caries experience (DMFT) in a convenience sample of adults in Sierra Leone, 2017 (*n* = 45).

Demographic items	Decayed teeth (DT)								Missing teeth (MT)			Dental caries experience (DMFT)									
	D_2–6_T		D_3–6_T		D_4–6_T		D_5–6_T					D_2–6_MT		D_3–6_MT		D_4–6_MT		D_5–6_MT		D_6_MT	
	Mean	%	Mean	%	Mean	%	Mean	%	Mean	%	*n*	Mean	%	Mean	%	Mean	%	Mean	%	Mean	%
Overall (*n* = 45)	12.80	100	11.9	98	6.8	98	3.8	80	1.4	42	65	14.2	100	13.4	98	8.2	98	5.2	84	4.0	78
**Sex**																					
Female (*n* = 29)	11.90	100	10.8	97	5.7	97	3.3	79	1.3	34	38	13.2	100	12.0	97	7.0	97	4.6	83	3.3	76
Male (*n* = 16)	14.20	100	14.1	100	8.8	100	4.6	81	1.6	56	27	15.9	100	15.8	100	10.5	100	6.2	87	5.3	81
**Area**																					
Urban (*n* = 16)	13.90	100	13.3	100	6.3	100	3.5	81	1.6	50	26	15.5	100	14.9	100	7.9	100	5.1	87	3.8	75
Rural (*n* = 29)	12.10	100	11.2	97	7.1	97	3.9	79	1.3	38	39	13.4	100	12.5	97	8.4	97	5.2	83	4.1	79
**Region**																					
East (*n* = 19)	14.60	100	14.5	100	9.3	100	4.5	84	1.1	37	21	15.6	100	15.5	100	10.4	100	5.5	89	3.4	79
South (*n* = 18)	13.70	100	12.8	100	5.3	100	3.2	72	1.1	44	20	14.8	100	13.8	100	6.4	100	4.2	78	3.6	72
West (*n* = 8)	6.30	100	4.1	87	4.1	87	3.4	87	3.0	50	24	9.2	100	7.1	87	7.1	87	6.3	87	6.3	87
**Ethnicity**																					
Mende (*n* = 27)	14.20	100	13.4	100	7.0	100	3.6	70	1.1	44	31	15.3	100	14.5	100	8.1	100	4.7	78	3.8	70
Temne (*n* = 8)	10.40	100	9.4	100	7.3	100	5.4	100	1.5	37	12	11.8	100	10.8	100	8.7	100	6.8	100	5.3	100
Others (*n* = 10)	10.80	100	10.0	90	5.9	90	2.8	90	2.2	40	22	13.0	100	12.2	90	8.1	90	5.0	90	3.6	80
**Age groups (years)**																					
18–24 (*n* = 4)	13.50	100	13.2	100	7.7	100	4.2	75	0.25	25	1	13.7	100	13.5	100	8.0	100	4.5	75	2.0	50
25–34 (*n* = 16)	13.30	100	12.5	100	6.9	100	3.8	87	1.3	31	21	14.6	100	13.7	100	8.2	100	5.1	94	4.0	88
35–44 (*n* = 20)	12.15	100	11.6	100	7.0	100	3.9	80	2.0	60	40	14.1	100	13.6	100	9.0	100	5.9	85	5.1	80
45+ (*n* = 5)	12.80	100	10.8	80	4.6	80	2.4	60	0.6	20	3	13.4	100	11.4	80	5.2	80	3.0	60	1.4	60

Note: There were no filled teeth (FT) across all participants.

Eighty-four per cent of examined participants had cavitated dental caries and visible dentine involvement (D_5–6_MT) with an average of 5.2 affected teeth per adult. Although the mean number teeth having dental caries experience at different thresholds was slightly higher in males as compared to females, it was not found to be statistically significant (Table 4).

Dental caries experience (DMFT) was slightly higher in the 25–34 years and 35–44 year age groups compared to younger (18–24 years) and older (45+ years) participants, with a mild decreasing trend in the number of decayed teeth with age; however, these differences were not statistically significant.

The participants in urban areas had similar proportions of dental caries experience; however, the mean number of DMFT was slightly higher at D_2–6_MT and D_3–6_MT in those from urban areas while it was slightly higher in rural areas at D_4–6_MT and above. Similarly, the mean number of DMFT was higher in the Western region at D_4–6_MT and above threshold, along with the average missing number of teeth when compared to Eastern and Southern region (Table 4). These differences at area (urban or rural) and regional levels were not statistically significant (*p* > 0.05).

### Presence of pain and sepsis as part of the pulp, ulcer, fistula, abscess examination

Half of the participants (51%, *n* = 23) who underwent clinical examination reported pain in their tooth/teeth. Forty-seven per cent (*n* = 21) had PUFA lesions, and all were of pulpal origin, with a mean of 1.2 teeth, which increased to 2.5 teeth in participants having PUFA score > 0.

### Proportion of non-carious conditions

Eighty-four per cent (*n* = 38) participants were found to have visible calculus in the oral cavity. Only two participants were seen to have suffered trauma to teeth involving pulp and reported to have a mucosal lesion (involving lips and buccal mucosa), respectively. Similarly, only two participants had upper dentures, and there was no fluorosis present in those who underwent oral examination.

## Discussion

The findings of the questionnaire survey revealed a high level of perceived need among participants, despite some positive health behaviours, notably widespread use of toothbrushes (97%) and toothpaste (93%). Over three-quarters (75%) had never visited a dentist, and nearly 40% reported toothache in the past 3 months. Urban participants were more likely to report good dental health (63%), whereas between 17% and 29% of rural participants experienced significant negative impacts on daily life due to oral health problems, including difficulties with eating, sleeping and social interaction. Younger participants (25–34 years) most frequently reported difficulty in smiling and relaxing or sleeping, while middle-aged participants (35–44 years) reported more difficulty in speaking. Daily sugar consumption was high, with 68% consuming at least one sugary item per day and males more likely than females to report sweet consumption. Clinical assessment revealed that all participants (*n* = 45) had experienced dental caries (D_2_ and above), with 84% having cavitated dental caries involving dentine (D_5–6_) and visible calculus. These findings underscore the substantial proportion of participants with untreated dental issues and the consequential impact on their oral health and well-being.

### Oral health behaviour and quality of life

Considering the dearth of oral health services in SL, it was not surprising that 75% participants reported that they had ‘never been to a dentist’, which was similar to findings reported through the CDHS-SL (over 66% of children).^22^ Similarly, most participants (> 90%) reported using a toothbrush and toothpaste, as also indicated by schoolchildren in SL (> 95%).^22^ Brushing twice daily was significantly more common in the Western region, likely due to better access to oral hygiene products and greater oral health awareness, supported by the presence of the country’s entire dental workforce.^4^ This contrasts with findings from other African contexts, such as Ethiopia, where between 12.2%^31^ and 19.3%^32^ of participants reported brushing twice daily, and Uganda,^33^ where the proportion was 56.5%.

Although the clinical findings of the participants who underwent a clinical examination indicated a high proportion with visible calculus (84%), there are no recent published data available from SL. However, the study by Normark from 1991^13^ reported 94% (*n* = 100) of adults between the ages of 35–44 years having calculus and pockets (CPITN-index). In addition, the CDHS-SL also reported 85% of schoolchildren (aged 12 years and 15 years) having supragingival (visible) calculus.^22^ A study from a comparable setting in Tanzania showed a similar trend, with most adults (94.6%, *n* = 159) involved in the oral health survey reporting that they brushed their teeth using a toothbrush and toothpaste and consumed a diet high in sugar (85.1%, *n* = 143; three times a day).

The role of diet and sugar on oral health is well documented.^34,35,36^ In this study, dietary and lifestyle behaviours varied significantly by sex and region. Females reported higher fruit intake, while males more frequently consumed sweets, smoked and used alcohol. Regional differences were also evident, with the Western region (which includes the capital Freetown) showing the highest fruit consumption and the Southern region reporting more frequent intake of cakes, biscuits and alcohol. Over two-thirds of participants (*n* = 82) reported consuming a sugary item at least once daily, in contrast to findings from Ethiopia, where only 10.7% of adults (*n* = 67) reported consuming sweets even once per week.^32^ The concern of easy availability and consumption of sugary diet in SL especially after civil war was reported by health professionals.^4^ The data from CDHS-SL showed 40% of schoolchildren consuming a sugary item at least once a day.^22^ Thus, the evidence suggests that while a high proportion of populations from sub-Saharan region report regular oral hygiene practices, other risk factors such as sugar, tobacco and alcohol are present and the burden of gingival and periodontal disease appears to be on the rise.^37,38^

Significant sex and regional differences were also observed in risk behaviours, including smoking and alcohol use. Male participants were markedly more likely than females to report both behaviours, consistent with findings from other African contexts.^39^ Alcohol use was significantly higher in the Southern region; however, the reasons for this pattern remain unclear. No notable environmental or infrastructural differences were observed between regions during fieldwork, suggesting other factors outside the scope of this study influencing it.

Considering OHRQoL, participants from urban areas reported more favourable dental health compared to their rural counterparts. Rural participants experienced a higher perceived burden on OHRQoL, with significant impacts related to difficulty in speaking, eating, cleaning teeth, sleeping, emotional well-being and social interactions. This is in line with findings from similar studies in other African settings, where rural participants experienced significantly higher proportion, intensity and extent of psychosocial impacts related to oral health conditions.^40^ Age-related differences were also evident, with difficulty in speaking most commonly reported among participants aged 35–44 years, whereas difficulty in smiling and relaxing was reported among those aged 25–34 years. These patterns align with findings from other African contexts, where younger people report greater concern about the social and aesthetic impacts of oral health problems.^41,42^

### Dental caries experience

All participants (*n* = 45) who underwent a clinical examination had extensive dental caries experience in more than five teeth (mean D_5–6_MFT = 5.2), just over half reported pain (51%, *n* = 23) and almost half had one or more PUFA lesions (47%, *n* = 21: pulpal origin). The proportion of missing teeth (M) was slightly higher in males (56%, *n* = 16) than females (34%, *n* = 29). In addition, 40% (*n* = 62) of all participants who responded through the survey reported to have experienced tooth decay in the past 3 months. These findings demonstrate the high burden of untreated dental caries experience, which was also highlighted in the qualitative study where Sierra Leonean dental professionals expressed their concern that people tend to neglect their oral health and often choose urgent treatments like extractions.^4^ Recently, a similarly high burden of dental caries experience was reported from amongst adults in Ethiopia.^32^ In addition, the CDHS-SL reported high levels (above 82%) of clinical dental caries experience (D_2–6_MFT) in school-going children across all four regions of SL.^22^

### DMFT threshold

This study used the ICDAS epidemiological tool, currently referred to as the ‘International Caries Classification and Management System (ICCMS)’, which has the advantage of enabling comparisons with earlier surveys conducted using WHO methodology.^43,44,45^ Based on ICDAS categories, the mean DMFT score could be described whether there is a ‘visual change in enamel’ (mean = 14.2, D_2–6_MFT), or ‘distinct decay into dentine’ (mean = 5.2, D_5–6_MFT), to ‘extensive decay into dentine’ (mean = 4, D_6_MFT) (as depicted in Table 4). The challenge, however, is that the oral health (including DMFT) data from countries such as SL is very limited. The last caries experience data on adults aged 35–44 years from SL published through WHO reported an average score of 5.3.^46^ Nevertheless, there is inadequate data from the WHO report to clarify the caries threshold. Various studies have focused on this challenge of establishing the caries threshold for comparison with ICDAS categories,^47,48,49^ but there is no agreement, and this issue is open to debate unless thresholds are clearly measured and reported.

### Strengths and limitations

Given the large young population in SL and the practicality of conducting nationwide research, our main epidemiological survey concentrated on child oral health.^22^ Therefore, a convenience sample to explore perceived and normative needs in adults was preferred given its simplicity and cost-effectiveness, recognising that it may not be representative of adult population in SL. As the survey was conducted along with the CDHS-SL,^22^ most participants were either parents of schoolchildren participating in the CDHS-SL or were present at the school site or anticipated dental treatment as a part of participating in the survey. Therefore, it was important to secure appropriate informed consent, which was accomplished by using a simple ‘pictorial information and consent (PIC)’ sheet developed with an illustrator.^27^ Moreover, the local SL team, who spoke all the main local languages, assisted in conveying information about the study to participants. In addition, given the dearth of recent adult oral health data,^13^ the research team deemed it a vital opportunity to look at adult oral health at least through the lens of a convenience sample.

Other possible limitation could be the low literacy levels, which could hinder participants to respond to a self-completion questionnaire in English. However, the local SL team proficient in local SL languages assisted any participants who requested support in completing the questionnaire. Nevertheless, there is a possibility of a bias as the participating adults could have been influenced either by other participating adults completing the survey or local SL team member assisting them. Furthermore, over 20% of cells in the chi-square tests had low expected counts (< 5), and although LR tests were used as an alternative, the results should be interpreted with caution. Additionally, as all participants who underwent clinical examination had some level of dental caries experience, it was not possible to assess associations based on caries presence versus absence. To address these limitations, future research should recruit larger and more demographically balanced samples to support robust comparisons informed by this study.

The unique strength of this study was showcasing the potential of ICDAS tool in a low-resource environment, allowing for the estimation and publication of oral health workforce requirements.^11^ This is important for SL, which has a severe shortage of oral health workforce with the dental professional to population ratio at 1:375 000,^11^ which is well short of the African regional average of 1:18 301.^50^ The national schoolchildren oral health survey (CDHS-SL) used operational research to estimate the population’s oral health needs and the pragmatic workforce required to deliver appropriate care.^11,22^ Therefore, it is vital for the future research to explore oral health needs of adults on a national level similar to CDHS-SL, which will not only validate the workforce needs projection for the general population but also inform realistic capacity-building as a part of the oral health strategy for SL.^21^

## Conclusion

This exploratory survey revealed considerable proportion of untreated dental diseases and poor quality of life among adult participants who participated in the clinical epidemiological examination, together with limited access to dental care and high behaviour risk. The findings underscore the need for a national adult dental health survey representative of adult population to further enhance planning of oral health services in SL in support of the national strategy.
